# Epidemiological characteristics of infectious hematopoietic necrosis virus (IHNV): a review

**DOI:** 10.1186/s13567-016-0341-1

**Published:** 2016-06-10

**Authors:** Peter Dixon, Richard Paley, Raul Alegria-Moran, Birgit Oidtmann

**Affiliations:** Centre for Environment, Fisheries and Aquaculture Science, Weymouth, Dorset, UK; Faculty of Veterinary and Animal Sciences, University of Chile, Santiago de Chile, Chile

## Abstract

Infectious hematopoietic necrosis virus (IHNV, *Rhabdoviridae*), is the causative agent of infectious hematopoietic necrosis (IHN), a disease notifiable to the World Organisation for Animal Health, and various countries and trading areas (including the European Union). IHNV is an economically important pathogen causing clinical disease and mortalities in a wide variety of salmonid species, including the main salmonid species produced in aquaculture, Atlantic salmon (*Salmo salar*) and rainbow trout (*Oncorhynchus mykiss*). We reviewed the scientific literature on IHNV on a range of topics, including geographic distribution; host range; conditions required for infection and clinical disease; minimum infectious dose; subclinical infection; shedding of virus by infected fish; transmission via eggs; diagnostic tests; pathogen load and survival of IHNV in host tissues. This information is required for a range of purposes including import risk assessments; parameterisation of disease models; for surveillance planning; and evaluation of the chances of eradication of the pathogen to name just a few. The review focuses on issues that are of relevance for the European context, but many of the data summarised have relevance to IHN globally. Examples for application of the information is presented and data gaps highlighted.

**Electronic supplementary material:**

The online version of this article (doi:10.1186/s13567-016-0341-1) contains supplementary material, which is available to authorized users.

## Table of contents

1 Introduction

2 Aetiological agent, agent strains

3 Geographic distribution of the pathogen

4 Host range

5 Host infection

6 Environmental conditions suitable for infection and clinical disease

7 Exposed host population susceptibility parameters

7.1 Strain of salmonid fish and hybrids

7.2 Age and size of the fish

7.3 Rearing density

7.4 Stress

8 Minimum infectious dose

9 Virulence of IHNV strain

10 Subclinical infection

11 Time between pathogen introduction into an aquaculture site and detection

12 Prevalence

12.1 Prevalence in wild populations

12.2 Prevalence in farmed populations

*12.2.1 Fish level prevalence*

*12.2.2 Farm level prevalence*

12.3 Prevalence data from experimental studies

13 Shedding of the virus

14 Transmission via eggs

15 Diagnostic test performance

16 Pathogen load in fish tissues

17 Survival of IHNV in fish tissues

18 Vectors of IHNV

19 Conclusions

Additional file

## Introduction

**Figure 1:**
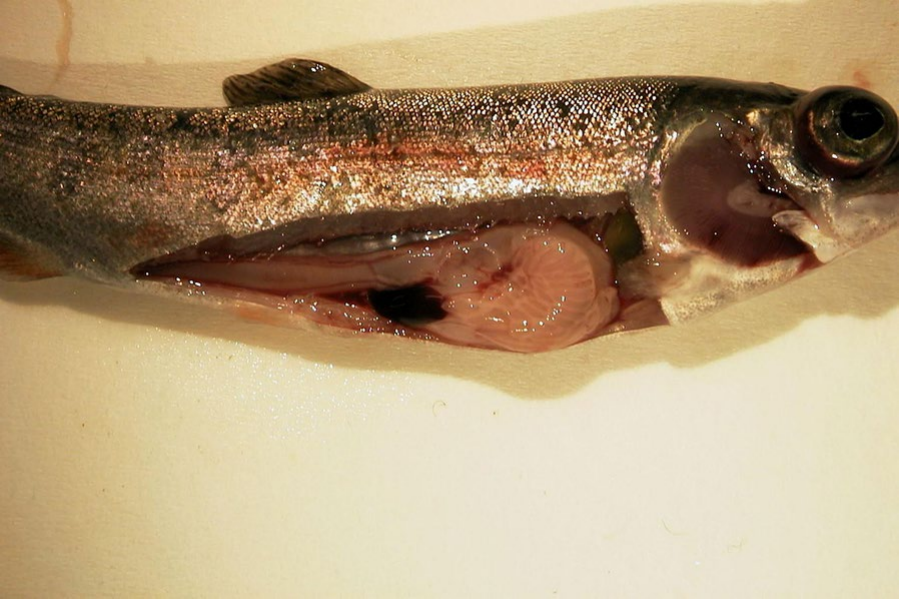
**Gross pathology of infectious hematopoietic necrosis in rainbow trout.** Typical gross appearance, including darkening of the skin, pale gills, exophthalmia, petechial haemorrhages, empty gut and ascitic fluid.

Infectious hematopoietic necrosis virus (IHNV) is an economically important pathogen causing clinical disease (Figure 1) and mortalities in a wide variety of salmonid species, including the main salmonid species produced in aquaculture, Atlantic salmon (*Salmo salar*) and rainbow trout (*Oncorhynchus mykiss*). In 2013, the worldwide production of all farmed salmonids exceeded three million tonnes, with a value of $17.5 billion [1]. Salmonid production, particularly Atlantic salmon, increased dramatically from 299 000 tonnes in 1990 to 1.9 million tonnes in 2010, at an average annual rate near 10% [2]. Infectious diseases are one of the main constraints to further expansion of aquaculture production [3]. Two epizootics of IHNV in Canada (from 1992 to 1996 and 2001 to 2003) caused a combined estimated economic loss to the salmon industry of CDN$40 million in inventory representing CDN$200 million in lost sales [4]. Infection of fish with IHNV is notifiable to the World Organisation for Animal Health, and various countries and trading areas (like the European Union) have particular legislation in place for the control of the disease. Initially identified in western North America, the pathogen spread to Europe and Asia [5, 6]. Data on the characteristics of a given pathogen are relevant for several purposes. In the epidemiological context such data are required for the preparation of import risk assessments (e.g. to evaluate the risk of introducing a given pathogen to support animal health policy with regards to trade and biosecurity); the parameterisation of disease models (e.g. to predict disease spread in the case of an introduction of the pathogen); to evaluate the chances of eradication of the pathogen; for surveillance planning (e.g. following a disease outbreak or to demonstrate freedom from disease) to name just a few.

The review focuses on issues that are of relevance for the European context, but many of the data summarised have relevance to IHN globally.

The scope of the review covers characteristics of the pathogen, the hosts and the likelihood of detection, all of which provide information towards the likelihood of pathogen transfer and establishment.

**Figure 2:**
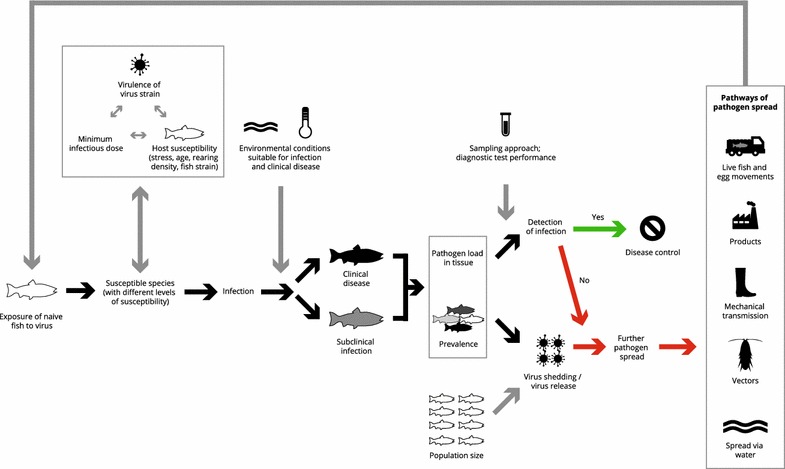
**Interacting epidemiological factors covered in this review influencing establishment of IHNV infection and disease spread**.

It is often relevant to understand some details of the studies referred to in order to evaluate the information provided and use it for risk assessments. For this reason, we provided information on the context and/or methods of the referenced papers. This makes this review a relatively detailed document; however, this was done with the view to provide a reference document that allows scientists to refer to the summarised (but not too summarised) information without necessarily having to refer to all the original sources. An overview of the interaction between the various epidemiological factors covered in this review and their impact on disease control is presented in Figure 2.

## Aetiological agent, agent strains

**Figure 3:**
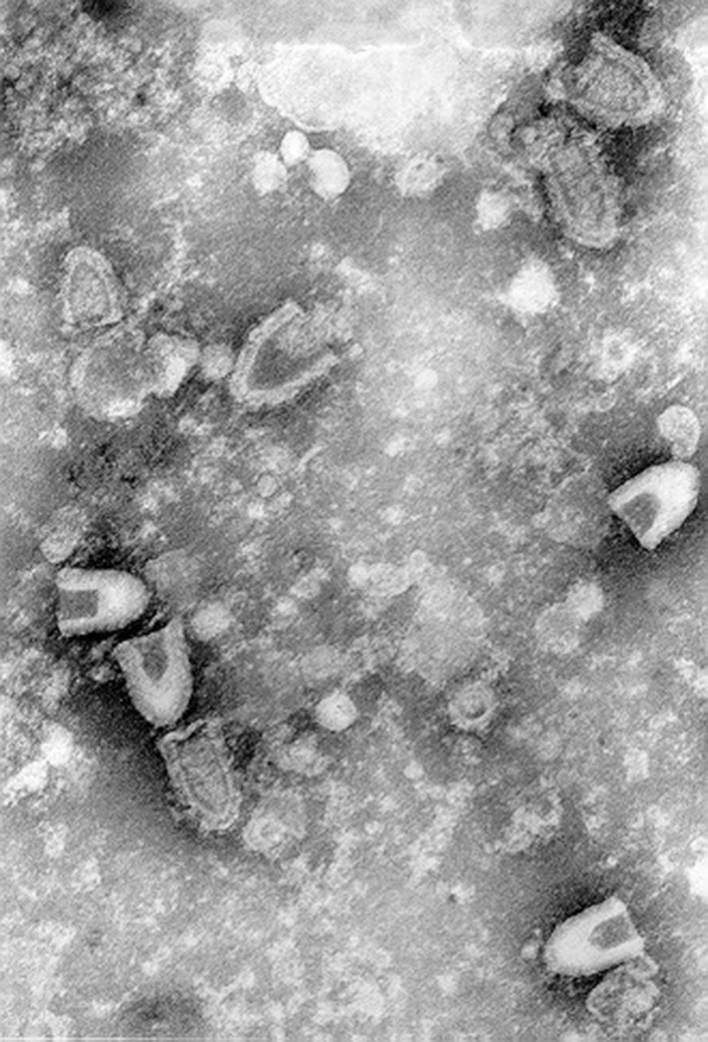
**Transmission electron micrograph of infectious hematopoietic necrosis virus (IHNV)**.

The causative agent of IHN, IHNV, is classified in the family *Rhabdoviridae*, and is one of three rhabdoviruses of finfish listed by the OIE (World Organisation for Animal Health). The IHNV virion is bullet shaped (Figure 3) and contains a single stranded, non-segmented, negative sense RNA genome of approximately 11 000 bases which encodes six proteins in the order nucleoprotein (N), phosphoprotein (P), matrix protein (M), glycoprotein (G), non-virion protein (NV) and polymerase (L). The NV protein is unique and its presence has resulted in the establishment of a separate genus, *Novirhabdovirus*, within the *Rhabdoviridae* with IHNV as the type species and the Western Regional Aquaculture Centre (WRAC) isolate (Genbank Accession L40883 for sequence) as the type strain. There appears to be one serotype in comparisons using polyclonal antisera [7], although sub-types/variants have been reported using monoclonal antibodies [8–10]. Different electropherotypes have been described (see Sect. 8), but the method currently most widely used for strain differentiation is through sequence analysis (e.g. [11]). Kurath et al. [11] designated three main virus genogroups [upper, middle and lower (U, M, L)] according, predominantly, to geographical range on the Western seaboard of North America. In general isolates from Pacific salmon form the U (sockeye salmon) and L (Chinook salmon) genogroups and display relatively limited genetic diversity indicative of historical evolutionary equilibrium whereas isolates from farmed rainbow trout in the USA form the M genogroup and show higher nucleotide diversity indicative of ongoing adaption to new host or conditions. The isolations from farmed rainbow trout in Europe and Asia (forming genogroups E and J) appear to have originated in the USA and their introduction into new environments with novel host species similarly leads to a high selection pressure on the pathogen, with very rapid evolution of IHNV [12, 13]. There appears to be no correlation between serotypes and genotypes [14].

## Geographic distribution of the pathogen

Initially identified in western North America, the pathogen spread to Europe and Asia. Historically, the virus has been reported from Austria, Belgium, Canada, Chile, China, Croatia, Czech Republic, France, Germany, Iran, Italy, Japan, Korea, The Netherlands, Poland, Russia, Slovenia, Spain, Switzerland, Taiwan and USA [15, 16].

According to the World Animal Health Information Database (WAHID–OIE, 2011 data) nine countries reported IHNV in domestic animals (fish produced in aquaculture) (Austria, China, Czech Republic, Germany, Italy, Japan, The Netherlands, Poland and Slovenia). Two countries reported cases in domestic and wild animals (France and USA) and one country reported the disease in wild animals only (Canada). The virus has recently been reported in the literature in wild fish in Kosovo [17] detected by RT-PCR only, and in cultivated fish in Turkey [18].

## Host range

Susceptible species are those from which the virus has been isolated or detected, but they do not necessarily exhibit IHN disease on a regular basis—if at all. Whether or not an aquatic animal species is susceptible to infection with a specific pathogen defines whether this species can potentially transmit the pathogen in question, either through trade of live animals or products (e.g. products for human consumption). Knowledge of the susceptible species range is therefore pivotal for containing disease spread and preventing its introduction into disease free areas. The listing of the susceptible species range is a core part of international trade regulations for aquatic animals (for trade into areas with a declared disease free status or undergoing programmes for disease eradication) [19, 20]. Knowledge of the susceptible species range is also relevant for targeting suitable species in surveillance programmes, e.g. to demonstrate freedom from infection or, in the event of an introduction of a given pathogen into a formerly disease free area, to establish its geographic spread. Species for which full or partial evidence for their susceptibility is available is summarised in Table 1.

**Table 1:** Species susceptible to Infectious hematopoietic necrosis virus

Scientific name	Common name	Listed as susceptible by EFSA [30]	Listed as susceptible by OIE diagnostic manual [16]	Disease commonly occurs/produces significant mortality
*Oncorhynchus mykiss*	Rainbow trout, steelhead trout	Yes	Yes	Yes
*Oncorhynchus tshawytscha*	Chinook salmon	Yes	Yes	Yes
*Oncorhynchus kisutch*	Coho salmon	Yes	Yes	Yes
*Oncorhynchus* *nerka*	Sockeye salmon, kokanee	Yes	Yes	Yes
*Oncorhynchus* *keta*	Chum salmon	Yes	Yes	Yes
*Oncorhynchus rhodurus*	Amago salmon	Yes	Yes	Yes
*Oncorhynchus* *masou*	Masu salmon	Yes	Yes	Yes
*Oncorhynchus* *clarki*	Cutthroat trout	Yes	(Yes)	
*Salmo salar*	Atlantic salmon	Yes	Yes	Yes
*Salmo trutta*	Brown trout	Not assessed	(Yes)	
*Salmo marmoratus*	Marble trout	Not assessed	Not listed	
*Salmo namaycush*	Lake trout	Yes	(Yes)	
*Salmo labrax* (syn. *Salmo trutta labrax*)^a^	Black Sea salmon	Not assessed	Not listed	
*Salvelinus alpinus*	Arctic char	Yes	(Yes)	
*Salvelinus fontinalis*	Brook trout	Yes	(Yes)	
*Salvelinus leucomaenis*	Char	Yes	(Yes)	
*Aulorhychus flavidus*	Tube-snout	Yes	(Yes)	
*Thymallus thymallus* ^a^	Grayling	Not assessed	Not listed	
*Esox lucius*	Northern pike	Yes	(Yes)	
*Plecoglossus altivelis*	Ayu	Yes	(Yes)	
*Acipenser transmontanus*	White sturgeon	Yes	(Yes)	
*Cymatogaster aggregata*	Shiner perch	Yes	(Yes)	
*Clupea pallasi*	Pacific herring	Yes	(Yes)	
*Gadus morhua* ^a^	Atlantic cod	Yes	(Yes)	
*Sparus aurata*	Gilthead seabream	II	No	
*Dicentrarchus labrax*	European seabass	II	No	
*Psetta Máxima/Scophthalmus maximus*	Turbot	II	No	
*Anguilla anguilla*	European eel	II	(Yes)	

II: scientific data partially support susceptibility, (Yes): described in OIE manual as “have occasionally been found to be infected in the wild or shown to be somewhat susceptible by experimental infection”.

^a^See comment in text.

In North America, IHN disease commonly occurs in sockeye salmon including the landlocked, kokanee form, Chinook salmon, rainbow trout (including steelheads) and Atlantic salmon [21]. Different strains of IHNV (identified in Sect. 2) are responsible for causing disease in these various species—this phylogenetic link between geographic isolation and species specific virulence is reviewed in Sect. 9. Where IHN occurs in Asia it is commonest in rainbow trout, chum and masu salmon [22–24]. In Russia the disease has only been recorded in sockeye salmon on the Pacific coast [25]. In Europe, IHN disease has only been recorded in rainbow trout [12]. The Atlantic salmon is a valuable cultured fish in certain European countries, but to date, IHNV has not been isolated from that species in Europe. Other species from which IHNV has been isolated in Europe, but usually without association with clinical disease, are the European eel [26, 27], Northern pike [28, 29], and brown trout [17]. Although not listed by either EFSA [30] or OIE [16], there is evidence suggesting that Black Sea salmon (*Salmo labrax*, syn. *Salmo trutta labrax*) [18], grayling (*Thymallus thymallus*) [31], and marble trout (*Salmo marmoratus*) [32] are also susceptible. Although Atlantic cod (*Gadus morhua*) is mentioned by both the EFSA report and the OIE diagnostic manual, the publication cited in the EFSA report [33] is based on an unnamed cod from the Pacific Northwest and hence is likely to be Pacific cod, *Gadus macrocephalus*, not Atlantic cod.

The identification of hosts not developing clinical disease are usually from single reports (and often as a side issue to the main subject of the report), which suggests that they are infrequent hosts and that significant mortality events in those species have not been observed.

These clear variations in the level of susceptibility of species to develop clinical disease have implications for planning of surveillance programmes, as clinical disease is usually associated with pathogen amplification [34]. For surveillance programmes, species most likely to develop clinical disease should be targeted, as this enhances the chance of pathogen detection. So for example in a fish farm that has both brown trout and rainbow trout, rainbow trout should be selected for sampling. However, when considering regulations, for example for control of live fish movements from infected farms, movements of species of low susceptibility (of low likelihood to develop clinical disease) would still need to be controlled as they pose a potential pathway of pathogen spread [35].

## Host infection

Information on host infection is relevant to inform suitable diagnostic test and sampling of suitable tissues for testing, but also when considering routes of transmission. The main mode of infection of fish with IHNV is via water or food; the potential for vertical transmission i.e. within the egg is discussed in Sect. 14. The primary portal of entry of IHNV has been considered to be the gills, but tissues of the digestive system may become infected e.g. if fish, particularly fry, that have died from the disease are eaten by others.

The course of infection has been followed in experimentally infected fish by a range of techniques including virus isolation, histology and immunohistochemistry. The general picture that emerges for rainbow trout fry and fingerlings is that virus is initially detected in gill epithelium, skin, the oral region, pharynx, oesophagus, stomach and pyloric caeca at 1–2 days post infection followed by detection in kidney, spleen, thymus, liver, muscle and cartilage at 3–4 days post infection; by day 5 post infection virus can also be found in heart, pancreas and brain [36–39]. Infectious IHNV was also detected in blood or kidney leucocytes of bath infected rainbow trout at 6 h post infection in 9% of samples, rising to 70% of samples positive by 18 h post infection [40]. IHNV can replicate in leucocytes cultured in vitro [40], but the authors suggested that the importance of leucocytes in IHNV infections was in spreading the virus throughout the body of the fish. In the studies cited above, mortalities typically commenced around 5 days post infection with a virulent IHNV isolate.

Studies using recombinant IHNV expressing the luciferase gene [41] showed that in bath infected 0.5 g rainbow trout fry infected with 5 × 10^4^ plaque forming units (pfu) virus mL^−1^, replication of virus was detected at the fin bases at 8 h post infection (although luciferase expression was first detected 2 h post infection at an unspecified location). No significant luciferase activity was seen in internal organs in the first 2 days but at 3 days post infection, the most active replication was in the spleen and kidney. In an initial experiment, luciferase activity on day 4 post infection was in the oral cavity, oesophagus, cardiac stomach, pyloric caecae, kidney and spleen as well as in the dorsal fin. The N gene and G gene of IHNV were detected at 1, 4 and 6 days post infection in the dorsal fins of bath infected rainbow trout using a SYBR green real time RT-PCR [42].

All the above studies used rainbow trout as the target species, but there is no reason to suppose that the sequence of events following IHNV infection are substantially different in other host species.

The infection process can be very rapid. Immersion of juvenile Chinook salmon, mean weight 1.37 g, in 5.7 × 10^3^ pfu mL^−1^ IHNV for 1 min resulted in 30% infection with no mortality, but following a 10 min immersion in the same concentration of virus there was 70% infection and a low level of mortality (<10%) [43]. Immersion of the Chinook salmon in 5.7 × 10^4^ pfu mL^−1^ IHNV for 1 min also resulted in 70% infection and a low level of mortality.

The results above suggest that following exposure of fish to high virus concentrations, virus detection could be possible within a few days post exposure. The virus can be found in a range of tissues and internal organs appear to become infected at a relatively early stage and are therefore suitable for sampling. Information regarding the viral load in various tissues is provided in Sect. 16. However, other factors, such as exposure dose (see Sect. 10), will influence progression to clinical disease and further spread of infection within a given host population. Subclinical infection may lead to low virus concentrations in tissues and escape detection using routine diagnostic methods.

## Environmental conditions suitable for infection and clinical disease

Surveillance programmes, e.g. for demonstrating freedom from infection, should be designed in a way that maximises the likelihood of detection of the pathogen in question. The best conditions to detect infection is when clinical manifestation of infection is most likely. The clinical manifestation of IHNV infection is influenced by environmental conditions, including water temperature and salinity.

All outbreaks of IHN described to date from Europe and the majority of IHN outbreaks in North America occurred in freshwater. However, three major IHN epizootics have also been reported in Atlantic salmon held in sea net-pens in British Columbia, Canada [4, 44, 45]. The IHNV is endemic among populations of wild salmonids throughout much of the west coast of North America, but clinical disease is not usually reported in wild fish at the seawater stage [16, 46]. Susceptibility of Atlantic salmon at the seawater stage with development of clinical disease and mortalities has also been shown experimentally by bath infection and/or cohabitation with experimentally infected fish [4, 47]. The west coast of North America is outside of the natural range for Atlantic salmon.

Epizootics of acute IHN usually occur at water temperatures of 8–14 °C [16, 21] and have not been reported above 15 °C [48], although chronic outbreaks in Chinook salmon have been reported at temperatures above 17 °C [43]. Under experimental conditions (freshwater) infection with mortality was induced in fingerling rainbow trout (0.2–0.3 g) at temperatures between 3 and 21 °C [49] following immersion in 10^5^ pfu mL^−1^ IHNV. When holding rainbow trout and sockeye salmon above 15.5 °C before infection or moving fish to a minimum of 18 °C within 24 h after infection and maintaining them at that temperature for 4–6 days prevented or reduced mortality [50, 51].

Based on the available information, the best conditions to target susceptible freshwater fish species is when water temperatures are between 10–12 °C. In the marine environment, although IHNV has been detected in a range of species, the only species in which clinical disease has been described—and is therefore most suitable to target in the marine environment for surveillance—is Atlantic salmon.

## Exposed host population susceptibility parameters

Information about factors influencing host population susceptibility is relevant for estimating the likelihood of establishment of infection in exposed fish populations and for an understanding of which subpopulations within farms are most likely to develop infection and mortality or morbidity and should therefore be preferably be targeted in sampling programmes. This information can therefore be used to inform and design surveillance programmes, including risk-based surveillance. The information is also relevant to inform disease spread models, e.g. to simulate how quickly infection is likely to spread within a farmed or wild fish population.

Susceptibility to infection and disease depends on several factors, e.g. fish species (see Sect. 4), fish strain, life stage, and environmental conditions the animals are exposed to (including, stress and rearing density). Furthermore, fish susceptibility varies with viral strain (see Sect. 9). Since these factors can be present in multiple combinations and data are usually presented for one set of conditions, generalisations across species and conditions are difficult to make.

### Strain of salmonid fish and hybrids

Besides variability in susceptibility between species (see Sect. 4), a range of studies have demonstrated variability of susceptibility to IHNV between strains or family lineages of fish. The average mortality of fry (progeny of 16 families of sockeye salmon generated by individual pair matings) challenged with IHNV ranged from 52 to 98%; their susceptibility was suggested to be based on genetic differences [52]. In a similar study of rainbow trout from 22 different families (bath challenged with IHNV), mortality ranged from 65 to 100% in 1 g fish, from 33 to 90% in 8 g fish and 10–85% in 25 g fish [53]. Chinook salmon strains from Washington State were more susceptible at fry stage than strains from Alaska [54].

There have been a number of studies investigating resistance to IHNV in different clones or strains of rainbow trout. Gynogenesis was used to produce nine homozygous clones of rainbow trout from a domestic population of rainbow trout. The clones were bath challenged with IHNV and mortality ranged from 16 to 100% in fish of <1.0 g [55]. A similar wide susceptibility range was observed in bath challenged rainbow trout from 25 families having different growth rates [56]. Mortality ranged from 7.5 to 88.2%. Survival was not correlated with growth rate per se, but body weight at challenge (using fish of a similar age from the different families) and survival were significantly correlated. Different strains of rainbow trout also differ in their antibody response to IHNV [57], which might also affect survival.

Use of hybrid fish strains has been shown to increase disease resistance. Triploid rainbow trout crossed with triploid coho salmon were significantly more resistant to experimental bath challenge with IHNV than pure crosses of either diploid or triploid rainbow trout [58]. This was confirmed in a later study which compared diploid and triploid pure crosses and hybrids of rainbow trout, brook trout and coho salmon for their resistance to IHNV. Progeny of female rainbow trout × male brook trout and female rainbow trout × male coho salmon crosses were significantly less susceptible to bath challenge with IHNV than the pure rainbow trout cross [59]. Likewise, triploid rainbow trout × brown trout hybrids were more resistant to bath challenge with IHNV (3–7% mortality) than pure rainbow trout (80% mortality) [60]. However, crossing coho and Chinook salmon did not confer resistance to the virus [61].

There was significantly less mortality in the triploid brown trout female × lake trout male hybrid progeny than in rainbow trout after bath infection with, or injection of IHNV [62]. The emergence of strains of IHNV from the MD subgroup of the M genogroup infecting steelhead trout was investigated in Washington State between 2007 and 2011. The overriding factor affecting emergence was variation in susceptibility of different populations of steelhead trout, rather than differences in the virulence of the virus strains, although the latter probably did have an effect. The resistance to some extent was heritable. The genetic basis for resistance in pure species and crosses has been investigated by a number of authors [63–73].

In summary, significant variability in mortality or morbidity levels is likely between fish strains. Increased resistance in certain fish strains means that detection of clinical disease (following infection), and therefore detection of infection is less likely. Increased levels of resistance are also likely to be associated with lower pathogen levels in fish tissues, making detection through routine sampling less likely. Where on a farm highly susceptible strains are present alongside more resistant strains, the highly susceptible strains should be selected for sampling.

Although such studies are currently not available, we would hypothesise that variations in susceptibility are also likely to impact on virus shedding, and prevalence (both likely to be decreased in more resistant strains), which will influence the dynamics of disease spread within a farmed fish population—which is relevant for disease modelling.

### Age and size of the fish

Fish up to 2 months old appear to be the most susceptible to IHNV in natural and experimental infections, although adult fish may be infected without exhibiting clinical signs or mortality [48]. However, there have been occasional reports of mortality in older fish, such as 1 year old Atlantic salmon [44], 14–16 month old sockeye salmon smolts [74] and 2 year old kokanee [75]. Arkusch et al. [76] reported that sexually mature Chinook salmon experimentally infected with IHNV died within 14 days of infection.

The susceptibility of rainbow trout (Isle of Man strain) in relation to age and size (2.5–3, 15–20 and 40–50 g) was investigated by exposing the animals to six different IHNV isolates (five European and one North American) [27]. All isolates (varying in virulence) were detected in rainbow trout of all ages/weights for 28 days. The two most virulent isolates (both European) caused mortality in fish independent of their weight or age. Two other European isolates were virulent in fish up to a weight of 3 g, but caused no mortality in larger fish [27].

LaPatra et al. [77] exposed four different age/weight groups of rainbow trout (0.2–13.1 g; 25–170 days old) and kokanee (0.2–7.2 g; 45–210 days old) to four concentrations of two strains (electropherotypes, see Sect. 8) of IHNV by immersion to determine the relationship between host susceptibility and host age/size. Rainbow trout were susceptible to a type 2 strain of IHNV at all the age/sizes tested, but became less susceptible to the type 1 strain with increasing age/size. The 0.2 g (45 day) kokanee were equally susceptible to both virus strains based on LD_50_, but the other age/size fish were more susceptible to the type 1 strain. With increasing age/size, kokanee became refractory to the type 2 strain but appeared to have increased susceptibility to the type 1 IHNV.

Experiments to determine whether age and/or weight was the determinant in mortality of rainbow trout fry were reported by LaPatra [48]. Fry that hatched on the same date were fed according to three different regimes resulting in groups of fish that were the same age but of three different weights. There was no difference in the LD_50_ between the three groups following challenge with IHNV when they were 3 months old, but an increase in survival with an increase in weight at 4 months of fish age. When challenging fish of the similar weight (around 4.7 g), but different age (3 or 4 months), the older fish were significantly less susceptible than the younger fish; however, when the experiment was repeated with fish of 4 and 5 months and similar weight, the older fish were more susceptible to the virus, which led the author to the conclusion that age or size alone does not determine host susceptibility. Recent work by Overturf et al. [56] indicated that weight of fish at challenge was an important determinant of survival in young rainbow trout of a similar age.

To summarise, for most host species, a decrease in susceptibly with increasing age or weight has been described.

For planning of surveillance programmes in freshwater farms, the reviewed data indicate that IHNV induced mortalities are most likely in juvenile fish (below 20 g). As the highly susceptible life stages are also likely to carry high pathogen loads, juvenile fish should therefore be targeted for sampling. Although data are not fully comprehensive, there appears to be little or no correlation between fish size/weight and susceptibility in fish up to 20 g.

It should be noted that (possibly due to costs) experiments usually use small fish. Apart from studies testing virus levels in fish around spawning time, data for near to market size fish are largely absent. Many of the studies use mortality as the measure of outcome. Subclinical infection therefore is often not captured.

### Rearing density

Rearing density is likely to influence IHNV transmission within a population of susceptible fish, as naïve fish are at an increased chance of being exposed to relatively little diluted virus released by infected fish at increasing rearing density.

Rainbow trout (1.2 g) were cohabited with a single experimentally IHNV-infected donor rainbow trout for 11 days at rearing densities of 8, 4, 0.63, 0.31, 0.16, 0.08 and 0.012 fish L^−1^ (water temperature 16–17 °C). However, at the end of the experiment, IHNV could only be isolated from 26 of 33 donor fish. No transmission was observed at the two lowest rearing densities, even though their donor fish were IHNV positive. At higher rearing densities, there was a direct relationship between rearing density and IHNV prevalence. At the highest rearing density, out of 60 exposed fish per setup (five repeats), between 1 and 4 became infected within the 11 day test period. The authors speculated that high rearing density could affect disease prevalence by (1) causing a deterioration in water quality leading to stress and a deleterious effect on the immune system and/or (2) increasing the likelihood of contact between naive fish and pathogen or infected fish [78].

The above mentioned study provides valuable information in that it demonstrates that increasing rearing density increases the probability of transmission and that at low densities transmission may not take place at all. The low rearing densities tested by the authors would not be expected to be found under farming conditions; they are more similar to what might be found in wild fish populations. At the highest rearing density tested (8 fish L^−1^, which would be a low to normal rearing density for this size of fish), transmission was consistently induced to naïve fish in all five replicate groups. At lower rearing densities, transmission from donor fish to naïve fish appeared to be a far more random event. As there are several factors influencing host population susceptibility, the transmission efficacy may vary depending on circumstances. The above mentioned experiments [78] provide a reproduction number, R0, relevant for the specific setup used in the study. R0 is one of the most crucial parameters needed for disease modelling. Although the study mentioned has clearly provided valuable information, further collection of data—either through field studies or experimental work—are important to explore how R0 may vary under different circumstances.

### Stress

Stress caused by physical handling, acute environmental changes, and a range of other factors is known to decrease fish resistance to disease [48]. In adult sockeye salmon, prevalence of detectible IHNV infection and viral load in host tissues increased in IHNV endemic areas as the migration of fish upstream and the annual spawning event progressed [46, 79]. Other stressors that have been noted in association with IHN disease epizootics include reduced food supply or high fish density of sockeye salmon in a lake in Alaska [80], low dissolved oxygen or high iron levels in groundwater from a well used in early incubation of eggs or fry of chum salmon [81], and high fish density allied with low water flow may have increased virus levels in, and subsequent shedding from spawning sockeye salmon [82]. Increased copper concentrations led to an increased susceptibility to IHNV compared with control animals [83], probably because of an immunosuppressive effect. In another study, rainbow trout were exposed to polychlorinated biphenyls (immunosuppressive in mammals), without significant effect on susceptibility to IHNV [84]. Exposure of 5–6 month old Chinook salmon to the insecticides chlorpyrifos and esfenvalerate did not increase the susceptibility of Chinook salmon to an isolate of IHNV isolated from steelhead trout and in some trials actually reduced the mortality in IHNV- and insecticide-treated groups (4.7–11.4% cumulative mortality), compared with IHNV infected control groups (18.6–21.1% cumulative mortality) [85, 86].

## Minimum infectious dose (MID)

Information about MID is mainly relevant for risk assessments and disease modelling. In the context of risk assessments, the MID determines whether infection is likely to take place following exposure of a susceptible fish population to IHNV. This may be via a range of routes, such as introduction of virus via fomites or aquatic animal products (e.g. fish products imported for human consumption or contaminated eggs for aquaculture).

The MID combined with virus shedding rates could theoretically be used to model disease transmission, e.g. within a farmed fish population or with regards to downstream spread. In the farm level context, virus shedding rates in combination with MID might be used to estimate R0 (the reproductive number of disease transmission). Foreman et al. [87] used the combination of MID and shedding rate to model IHNV spread in Canada.

Despite IHN disease being known for over half a century, dedicated experiments to determine the MID are limited in the scientific literature. Early studies show infection with or without mortality at concentrations as low as 10^2^ pfu mL^−1^ [50, 88, 89]. In kokanee (0.2–7.2 g) and rainbow trout (0.2–13.1 g) cumulative mortalities of up to 88 and 100% respectively were reported following bath exposure challenges at the lowest concentration tested of 10^1.9^ pfu mL^−1^ [77]. Conversely, mortalities in fingerling rainbow trout (0.2–0.7 g) bath challenged with virus concentrations increasing on a log_10_ scale between 10^1^ and 10^5^ pfu mL^−1^, were observed from 10^3^ pfu mL^−1^ or higher [83]. MID was shown to vary according to the isolate used and the host species tested, following challenge of Chinook salmon and rainbow trout with IHNV isolates of different electropherotypes, and of different host and geographic origin [90]. Studies cited above tended to use mortalities as the outcome measure for susceptibility. Further studies demonstrated that asymptomatic infections can occur in Chinook salmon when fish are exposed for as little as 1 min to >10^2^ pfu mL^−1^. Progression to clinical disease was infrequent unless the challenge dose was >10^4^ pfu mL^−1^ [43]. Atlantic salmon smolt (average weight 122 g) reared in seawater of 10 °C were susceptible to infection at 10^1^–10^4^ pfu mL^−1^ (exposed for 1 h). Mortality was observed at all exposure concentrations, but only in one of duplicate tanks at the lowest dose. Variations in mortality rate were observed depending on exposure dose, and mean time to death was shorter in the higher challenge dose groups [4].

In summary, the MID appears to vary depending on fish species and for fry of the highly susceptible species to range between 10^2^ and 10^3^ pfu mL^−1^. In Atlantic salmon smolt, the MID was found to be even lower at 10^1^ pfu mL^−1^. As stated before, host susceptibility is likely to vary depending on a range of factors such as rearing temperature, stress and several more. Therefore, the MID may not be automatically applied to a field situation. It also makes its use in the context of disease modelling more complex, as exposure of susceptible fish at the MID does not necessarily result in infection. In fact, near the MID, infection is a random event, which means transmission to a susceptible host population only takes place in a fraction of exposures at this dose.

The above also shows that the duration of virus exposure is crucial. In field situations, exposure concentration is likely to vary and may-depending on circumstances—only last for short periods of time (e.g. wild infected salmon swimming close to an Atlantic salmon net cage; a batch of highly infected fish being processed and the liquid waste being discharged for a limited time period; first introduction of a contaminated fomite into an aquaculture facility).

## Virulence of IHNV strain

Isolates of IHNV differ in the molecular weight of major structural proteins, which enabled the separation of strains into four types (termed electropherotypes) following electrophoresis [91]. That was later expanded into five types to accommodate isolates that had been incompletely characterised [92]. LaPatra [93] reported differences in virulence for rainbow trout of IHNV isolates from different electropherotypes. Following bath infection in rainbow trout, type 1 and 3 strains (potentially L genogroup, see below) caused 4 or 6% mortality, whereas a type 2 strain (potentially M genogroup) caused 62% mortality. Further studies led to conflicting views as to the main factor affecting virulence: host [47, 77, 94, 95] or geographic origin of the virus [90].

Subsequently three genogroups of IHNV were described based on phylogenetic analysis of 303 field isolates from North America. The genogroups had three major geographic ranges in the Pacific Northwest, and were labelled Upper (U) for the northernmost group, Middle (M) and Lower (L) for the southernmost group [11]. These later studies support the hypothesis that geographic origin of the isolates played a greater role than host origin: Virulence of isolates of the three genogroups were compared by bath exposure of juvenile sockeye salmon, kokanee and two strains of rainbow trout. In sockeye salmon and kokanee the U genogroup strains (three tested) were highly pathogenic (69–100% mortality) whilst the M strains (three tested) were hardly pathogenic (0–4% mortality). However, the M genogroup strains were more virulent for rainbow trout (25–85% mortality), whilst the U genogroup was less virulent (5–41% mortality) for that species. The L genogroup (one strain tested) showed medium virulence to both sockeye and rainbow trout (13–53% mortality) [96]. These data suggest that genogroup virulence is related to geographic origin of the virus strain. Virulence for a particular species may also be related to the long- or short term association of a strain with a particular host. For instance, it has been hypothesised that a U genogroup strain (or strains) jumped from sockeye salmon to rainbow trout, evolving into the M genogroup and having greater virulence for the new host and losing virulence for the original one [11, 97]. The fitness of three pairs of IHNV M genotypes in the Columbia River basin was assessed by in vivo replication kinetics, host interferon-induced Mx-1 expression during single infections and the ability to replicate in co-infection and superinfection studies in steelhead trout [98]. Increased fitness did not correlate with displacement of one genotype by another in the field, suggesting that other factors were important for such displacement events, such as increased ability to shed into the water, leading to increased chance of transmission or increased ability to persist in the host leading to increased duration and distance of transmission.

In summary, there are clear differences in virulence depending on viral strain and species infected. Although it is not absolute, in general a strain isolated from a given species corresponding to its historical phylogeographic host tends to be more virulent for the same species (i.e. virulence lower in other species).

Differences in virulence of strains are mainly relevant for predicting the outcome of IHNV exposure: where highly pathogenic IHNV strains are present in the relevant host species, the impact in terms of mortalities would be expected to be significant. On the other hand, infection with strains in a less relevant host may go unnoticed and movements of infected fish undertaken, until contact with a species more susceptible to the particular strain or conditions more suited for clinical expression leading to detection.

## Subclinical infection

Subclinical infections play a particularly important role for the risk of spreading IHNV through live fish movements. Farmers may be unaware of infection in their stock and move fish to uninfected destinations. This is of particular concern if movements take place into disease free farms, zones or countries. Therefore, subclinical infections are of particular relevance for risk assessments, but also for disease spread modelling.

Subclinical infections tend to be associated with lower virus levels in affected fish compared to fish undergoing clinical infection [34], making detection through routine surveillance programmes difficult. As a result, this could lead to false assumption of freedom from infection.

Susceptibility to infection depends on a range of host factors, such as species and life stage (see Sect. 7) but also viral strain (Sect. 9) and environment (Section 6; Figure 2). The virus has been isolated from several fish species, but causes clinical disease associated with high mortalities only in a limited number of species, such as juvenile sockeye salmon, Chinook salmon, chum salmon, masu salmon, rainbow trout and Atlantic salmon [21–24, 44, 99], Mortalities, usually at a low level have occurred in cultivated brown trout [100, 101], but virus or evidence of virus has been detected in wild brown trout with no clinical disease in a number of countries [17, 102], leading to concerns as to the role of that species as a reservoir of the virus [17, 32]. Furthermore, subclinical IHNV infections have also been described in those species known to develop clinical signs, e.g. Chinook salmon [43], adult sockeye salmon [46, 79, 103], rainbow trout, [104] and Atlantic salmon [105]. In the more susceptible species and life stages, subclinical infections tend to occur when fish are exposed to low doses of the virus and/or for short time periods, as has been shown in Chinook salmon fry [43]. Progression to clinical disease was infrequent unless the challenge dose was >10^4^ pfu mL^−1^ [43].

Virus can be detected for months to over a year post exposure: in Chinook salmon IHNV was detected up to 39 days post exposure in subclinical fish after exposure to 1.9 × 10^3^ pfu mL^−1^ (isolate fCLChn-n6 isolated from fall-run adult Chinook salmon from the Coleman National Fish Hatchery, i.e. potentially L genotype) [43]. Persistence of IHNV has been detected in rainbow trout survivors 1 year after exposure as fry, using immunohistochemical, molecular biology and electron microscopy techniques, though infectious virus was no longer detectable by plaque assay beyond 46 days post exposure [106]. Another study detected presence of persistent IHNV in rainbow trout survivors 18 months post infection, using RT-PCR in liver and kidney samples [104]. In Sockeye salmon fry (average weight 5.5 g) bath infected with IHNV, which resulted in approximately 35% mortality over 4 months, virus was detected in the brains of survivors by reverse transcriptase real time PCR (RT-rPCR) 9 months post challenge [103].

Transmission of the virus has been demonstrated from asymptomatic post-smolt Chinook salmon to Atlantic salmon under experimental conditions [107]. Post-smolt Chinook salmon that had survived IHNV bath exposure passed infection on to Atlantic salmon as long as 5 months after the initial exposure, although transmission was more effective 22 days after the initial exposure [107]. At the end of the experiment (60 days after the Atlantic salmon were exposed to water from a tank containing asymptomatic, IHNV-infected Chinook salmon), none of the surviving Atlantic salmon were positive for IHNV but one of the surviving Chinook salmon was.

In areas where IHNV is endemic, the virus is generally rarely seen in adult fish except around the period of spawning [46, 79]. Clinical disease is uncommon in adult fish (see Sect. 7.2) and adult fish may carry the virus at undetectable levels until virus titres increase due to spawning stress. Infected wild fish populations rarely display clinical disease (e.g. [17]).

Further evidence for subclinical infection comes from experimental challenges, where susceptibility was demonstrated for a given fish species, but infection did not always concur with mortality or clinical signs [108]. Furthermore, IHNV was detected in non-clinical fish during surveillance programmes or surveys [34]. Therefore, species susceptible to infection but less likely to display clinical signs such as brown trout, brook trout and pike play a particular risk for unintended spread of the virus [35]. Similarly, life stages less prone to develop clinical signs (i.e. adult fish) may well be infected, but without detectable clinical signs [27]. Low virulent strains may lead to infection without causing clinical disease [27].

Overall, it is difficult to truly assess how common subclinical infection is, since due to limitations in diagnostic test sensitivity (see Sect. 15), subclinically infected fish may escape detection. Detection of antibodies against IHNV in fish may assist in the detection of carrier populations, as it indicates previous exposure. However, presence of IHNV antibodies is not necessarily indicting current infection with viable virus. Therefore, serological data would need to be carefully interpreted.

## Time between pathogen introduction into an aquaculture site and detection

The time period between pathogen introduction onto a site and detection is relevant when dealing with an ongoing disease epidemic (e.g. when trying to contain spread, or establish geographical spread of infection), and also for disease modelling, as measures to prevent further spread may only be taken once infection on farm is suspected. The time to detection depends on several factors, including: amount of virus introduced into a new farm; incubation time period; speed of increase in prevalence; the existence of long time carrier status, and the capability of diagnostic tests to detect infection.

Most of the data on clinical infections with IHNV come from the experience in North America. There, typically, returning Pacific salmon are spawned, the progeny are raised in hatcheries and that is the time when clinical disease in fry is often observed. Migrating Pacific salmonids are not a feature of European aquaculture, and pathogen introduction onto a site does not follow the same pattern. In Germany, and probably many other European countries, the main mode of transfer of IHNV is by trade in infected fish [12, 35].

Infection on a farm site can remain undetected for long periods of time and may even remain undetected. With the exception of Atlantic salmon, virus concentrations required to induce infection and clinical disease in exposed salmonids appear to be relatively high [4, 109]. On a farm site, this would mean that low level introductions would lead to a long delay before virus levels build up to levels leading to increased mortalities.

The much larger number of naive fish means that low level mortalities may not initially be picked up, until prevalence has reached relatively high levels. Adding to this is the difficulty of detecting dead or dying animals accurately. Exposure to low level concentrations (e.g. 10^2^ pfu mL^−1^ IHNV) often does not lead to detectable infection [43]; if it does, the virus may only be detectable for limited time periods [109]. Detection of the virus in fish long time periods after first exposure suggests that fish develop a carrier state, which escapes detection by routine diagnostic methods. Time periods between first exposure and re-emergence in individual fish can be as long as 1 year [106].

In outbreaks of IHN in Germany in 2006, time to appearance of noticeable change in mortality or appearance of clinical signs varied between a few weeks and 5–6 months. The long incubation periods occurred on farms that became infected in spring, when temperature had risen already to above 15 °C and infection only became clinically apparent when water temperature dropped again in autumn (E. Nardy, personal communication, March 2016).

## Prevalence

Estimates of prevalence are relevant for planning surveillance programmes or for interpreting surveillance data. Surveillance programmes usually target farmed animals, as results have implications for the ability of farms to trade. Knowledge of infection levels in wild populations is of relevance where spill over of virus from wild to farmed fish (or vice versa) is suspected.

### Prevalence in wild populations

In North America, IHNV is endemic among populations of wild salmonids throughout much of its historical range along the west coast of North America [16]. Studies in sockeye salmon have shown variability of detectable infection depending on life stage: juvenile sockeye salmon are fairly susceptible to infection [48]; between 1973 and 1999 the losses of juveniles as a percentage of eggs produced in Alaskan hatcheries ranged from 0 to 48.8% [110], although losses of up to 99% have been recorded [111]. In adult sockeye salmon, detectable prevalence is low or infection not detectable in fish returning from the sea to their spawning grounds (virus was found in 7 of 60 fish tested (12%) at only one of four marine sites tested), but increases substantially when fish reach the spawning site [up to 50% (30 of 60) of fish tested were IHNV positive; results based on IHNV isolation] [46]. Data from long term studies showed an average prevalence in wild populations of female spawning Alaskan sockeye salmon (based on testing ovarian fluid) of 40.4% (max 56%, min 8.5%; annual data from 1980 to 2000) and 53.6% (min 11.3%; max 60.3%, annual data from 1980 to 1988) in postspawned females [110]. The proportion of fish with high viral titres (≥10^4^) was on average above 40%.

In Europe, very few studies have investigated presence of IHNV in wild fish populations. In surveys undertaken in Switzerland in 1984, 1985 and 2000, IHNV was not detected in samples collected from wild fish populations (mainly brown trout). In total, tissue samples from near to 600 fish from natural habitats were tested by tissue culture. In addition, submissions of wild fish (*n* = 156 between 1978 and 2000) to the lab from reported disease incidents did not detect IHNV. IHNV was not detected on fish farms in Switzerland in the years of the wild fish surveys (1984, 1985 and 2000), but between 1993 and 1999 [112].

A study of free-ranging rainbow trout in Germany in the vicinity of farms that had either confirmed outbreaks of IHN or had introductions of fish from known infected sources less than 4 months prior to sampling reported a prevalence of 35% (15 out of 43 rainbow trout testing positive) based on detection of IHNV neutralising antibodies [113]. It is not clear from the paper, whether the 43 fish were sampled from multiple locations. Detection of IHNV neutralising antibodies indicates exposure to the virus but not necessarily current infection. Therefore, the proportion of fish with IHNV neutralising antibodies are more likely an indication of the cumulative proportion of fish with prior exposure.

In a study from five rivers in the Republic of Kosovo, wild brown trout were sampled. Of a total of 32 pools (of five brown trout tissue samples, mixed fish sizes), eight pools tested positive for IHNV by RT-PCR (25.0%). The positive pools were from 3 of the 5 rivers. No signs of disease had been detected in the sampled fish. Interestingly, IHNV was not detected in farmed rainbow trout from Kosovo during the study period (2006–2008). However, it was not reported whether the rainbow trout farms tested were located on the same rivers as the three from which brown trout tested positive [17].

As described above (Sect. 7.1), levels of susceptibility vary by species (e.g. rainbow trout are more prone to develop clinical disease compared to brown trout). Therefore, species with higher susceptibility would be more likely to test positive compared to less susceptible species. Furthermore, whereas IHNV is endemic in wild sockeye salmon populations on the west coast of North America, endemic infection in feral fish populations in Europe appear less common. This is likely to be a reflection of the susceptibility levels of feral fish species found in rivers.

### Prevalence in farmed populations

#### Fish level prevalence

Statutory sampling programmes usually recommend or prescribe pooling of samples (see Sect. 15). In consequence most results available from farm surveys are from pooled samples, which does not allow determining fish level prevalence. One of the few studies reporting results at fish level tested rainbow trout from 30 farms in Germany, most of which had a history of IHN or viral haemorrhagic septicaemia (VHS) or a current VHS/IHN outbreak. IHNV was detected in 7 of the 30 farms by RT-PCR, but only in three farms using virus isolation by cell culture. Of the fish tested from the seven IHNV positive farms, 59% (23 of 39 fish) tested positive by nested RT-PCR. At least one fish per farm tested positive by nested RT-PCR. PCR results were not broken down by number of fish positive against individual farms. Results of virus isolation are not reported by individual fish. The farms testing positive by RT-PCR but not by virus isolation had subclinical IHNV infection [34].

The dynamics of IHNV infection and disease were followed in a juvenile Chinook salmon population from North America both during hatchery rearing and for 2 weeks post-release. Cumulative weekly mortality increased from 0.03 to 3.5% as the prevalence of viral infection increased from 2 to 22% over the same four-week period. The majority of the infected salmon were asymptomatic [43].

In adult Atlantic salmon from farmed populations that had experienced an outbreak of IHN a year before samples were collected, no live virus could be isolated, but more than 60% of the fish were positive for antibodies against the virus [105]. An IHN epidemic that occurred in Atlantic salmon over almost 2 years in British Columbia has been analysed [114]. Thirty-six farms were affected but a further 19 farms in the region were not. Over 12 million Atlantic salmon died or were culled during the epidemic, and losses on individual sites holding salmon for at least 14 weeks during the outbreak ranged from 20 to 94%; higher mortalities occurred in populations that had been in seawater for <1 year (smolt populations) with reduced mortality in older populations.

No published data are available following prevalence of infection during the course of a clinical outbreak on farms in Europe.

As described above, host-factors (Sect. 7), environmental conditions (Sect. 6) and pathogen factors (Sect. 9) will influence the course of the disease (Figure 2), and in result detectable prevalence. These factors may vary between rearing units within a farm and therefore sampling programmes should target rearing units in which detection of the virus is most likely. Furthermore, prevalence obviously also depends on the stage within an outbreak. During the course of a clinical outbreak (depending on number of infected fish and/or infectious dose initially introduced into the farm) prevalence would be expected to increase from very low (well below 1%) to considerably higher levels (possibly in excess of 50%).

#### Farm level prevalence

The National Reference Laboratories for fish diseases in Europe provide reports on detection of IHNV on fish farms [115].

Out of 36 countries that provided data, seven considered one or more farms in their territory as IHNV infected in 2010. Amongst countries which reported IHNV on farms, the number of farms considered to be infected ranged from 1 (Belgium, Czech Republic) to 73 (Italy). Several countries had a considerable number of farms, for which the IHNV status was unknown. Therefore, farm level prevalence data cannot be derived.

Data for 2014 show a considerable change compared to 2010. Only one farm in Italy (out of 901 farms holding susceptible species) was considered to be infected with IHNV, and in Slovenia 28 (of 321) farms. Further countries reporting presence of the disease were Austria (1 farm), Belgium (1 of 90 farms), Croatia (4 of 304 farms), France (1 of 1622 farms), Germany (11 of 15 812 farms), The Netherlands (8 of 59 farms), Poland (3 of 4442 farms) [116].

The picture that emerges for Europe is that—in contrast to the situation on the West coast of North America—IHNV is probably not endemic in wild fish populations. Detection of the virus in wild fish are most likely due to spill over of virus from farmed into wild fish populations, which—once the source of virus spill is removed—appear to eliminate the virus over time. Introductions of the virus into previously virus free farms, are most likely from infected farms and not from the wild.

### Prevalence data from experimental studies

Juvenile rainbow trout and sockeye salmon exposed to two different strains of IHNV (U and M strain) showed clearly different prevalences and mortalities depending on species and viral strain [117]. No mortalities were observed amongst the sockeye salmon, whereas mortalities were observed in most of the rainbow trout groups (72 h observation period). Prevalence was near to or 100% in both species when exposed to the M strain in a single infection. The U strain produced 100% prevalence in sockeye salmon (single infection), but only up to 18 out of 30 rainbow trout tested IHNV positive.

In an experimental study, prevalence (IHNV infection) in rainbow trout following exposure to IHNV infected rainbow trout ranged from 0 to 19% within 6 days post exposure (average fish weight ca. 1.2 g) without clinical manifestation of the disease in the cohabitated fish within the 6 day observation period [109].

In conclusion: fish level prevalence depends on a number of factors including host species, life stage, IHNV strain, and stage within an epidemic (Figure 2). Very few data on fish level prevalence are available from farmed fish. Several susceptible species may carry IHNV in a clinical inapparent infection and in these circumstances, infection may not be detected by some diagnostic tests. Therefore for the planning of surveillance programmes and interpretation of surveillance data will need to take into account the factors affecting fish level prevalence. An overview of factors to consider in the design of a sampling programme is provided below (Sect. 19).

## Shedding of the virus

Data on viral shedding rates per fish or biomass per time unit are essential to predict virus concentrations in the environment and as a result exposure levels for naïve fish cohabited with infected fish or exposed to virus contaminated water. Data on shedding rates per fish need to be combined with prevalence data to extrapolate shedding rates from a given fish population. These again will need to be put into context with water flow rates, which will dilute virus concentrations—possibly to levels below the minimal infectious dose.

Knowledge of virus excretion pathways and virus levels found in these fish excretions is relevant as they present the routes of pathogen release, which may have relevance for transmission pathways (e.g. egg associated transmission, see Sect. 14).

IHNV is shed in the external mucus and sexual fluids of fish [76, 110, 118–121]. It has occasionally been detected in faeces [122], but virus shedding via this route seems to be rare and of little relevance overall [121]. Some publications state that IHNV is shed in urine, but these authors have been unable to find any data in the scientific literature to substantiate that claim.

In juvenile fish undergoing an acute outbreak, the most relevant shedding route is therefore via mucus. In rainbow trout fry (mean weight 2.4 g) the virus was detected in mucus from 24 h after water borne exposure and increased to levels near 10^4^ pfu mL^−1^ by 48 h post exposure, remaining at this level for at least 2 days. The virus was not detected 12 h following exposure, suggesting that virus was not contamination from the initial bath challenge. Virus concentrations were even higher (10^3.6^–10^6.0^ pfu mL^−1^) in mucus of kokanee salmon and steelhead trout (mean weight 0.8 g), which had died following IHNV exposure. Similarly high concentrations were found in Chinook salmon (mean weight 1.8 g) that had died following natural infection. Virus levels were slightly lower in yearling Chinook salmon with chronic infection (10^1.3^–10^2.4^ pfu mL^−1^) and adult steelhead trout (male and female) in spawning condition (mostly around 10^2^–10^3^ pfu mL^−1^) [118]. Unfortunately, no data were provided on concentration of virus in rearing water in the days post challenge. Furthermore, rates at which the mucus is shed from fish per time unit and with it virus are not known. Therefore, shedding rates per time unit cannot be derived.

Several studies have reported virus concentrations in reproductive fluids. Virus levels range from 0 to 10^10^ pfu mL^−1^ [76, 120] showing significant variation within populations (temporal trend) and between populations [120]. Virus levels tend to be higher in ovarian fluid compared to those in milt and prevalence tends to be higher in females compared to males [119–121]. Prevalence and virus levels were found to be higher in post spawning sockeye salmon (8 and 26 days post spawning) compared to spawning fish [110]. No data are available on rates of release of sexual fluids. Therefore, actual quantities of virus released via this route remain unclear.

A range of studies investigated IHNV levels in rearing water during IHN outbreaks in juvenile fish or from water holding adult fish around spawning time. Virus levels in water from holding channels of female sockeye salmon were up to peaks of 1600 pfu mL^−1^ for postspawning sockeye salmon, but were lower earlier on in the spawning period (in the order of 30–400 pfu mL^−1^) [82]. The extremely high titre of 1600 pfu mL^−1^ may have been caused by a disturbance of the sediment that took place prior to sampling leading to release of virus from the sediment, or by release of virus from decomposing spent salmon in the water [82].

Levels of 0.02–0.2 pfu mL^−1^ were detected in effluent from an adult steelhead holding pond, and 1 pfu per 3 L water from a river [123]. The amount of IHNV detected in river water in Japan was 0.56 50% tissue culture infectious doses (TCID_50_) per L, and 5.60 TCID_50_ per L was detected in pond water in which there was an outbreak of IHN in rainbow trout fry [124]. In another study, virus titres were recorded in water before and during an IHN epizootic in a steelhead trout rearing facility. Virus was not detected in the hatchery water supply or in adult fish holding ponds. In the early stages of an IHN outbreak when mortalities were low (<20–250 per day), median levels of virus in nursery tank water were 0.2–0.5 pfu mL^−1^ increasing to 3 pfu mL^−1^ when daily mortalities were 500–2000. Levels of 5–50 pfu mL^−1^ were recorded in rearing ponds that had received fry from the nursery tanks [125].

As rearing density and flow rates were not provided, shedding rates cannot be extrapolated from the above studies.

A study investigating release of virus from juvenile Chinook salmon showing clinical signs of IHN during an epizootic [43] reported immediate (after 1 min) high levels (mean concentrations of near 1000 pfu mL^−1^; range: 50–2500 pfu mL^−1^) of virus following transfer of infected individual fish into eight separate static 100 mL containers at 13 °C. Similar mean concentrations of 1000 pfu mL^−1^ were also seen after 10 and 30 min (with a similar range to that at 1 min). Fish mucus samples contained 6 × 10^4^–2 × 10^7^ pfu mL^−1^ [43]. The immediate detection of high virus levels following placing of the fish in the container, which then remain relatively unchanged over the 30 min observation period, suggest that initial increase is due to initial mucus shedding—possibly due to transfer stress. This information is highly relevant, as it suggests that when fish are moved, there is an initial burst of virus release. Therefore, one should not necessarily assume a constant rate of virus shedding immediately after fish movements or stressful handling events that may be associated with mucus shedding. This is very significant, as it is this particular burst of virus shedding that could bring virus levels in a farm locally to levels where the MID is reached.

The first study to actually provide shedding rates over a longer time period was investigating the release of virus from IHNV infected Atlantic salmon. IHNV was detected before the onset of visible signs of disease with peak shed rates averaging 3.26 × 10^7^ pfu fish^−1^ h^−1^ one to two days prior to mortality. Onset of shedding in individually reared fish was observed at the earliest on day 9 after bath exposure (not all of the bath challenged fish started to shed virus during the 3 week observation period), and at the earliest on day 5 in salmon injected with the virus. Shedding continued in some fish until the end of the 3 week observation period [4].

Using the data published by Garver et al. [4] shedding rates from marine Atlantic salmon farms during the peak rate of shedding for a farm of 500 000 fish was estimated at 1.6 × 10^11^ and 3.2 × 10^9^ pfu fish^−1^ h^−1^ for a vaccinated population [87].

## Transmission via eggs

Knowledge of likelihood of transmission of IHNV via eggs is relevant in the context of risk assessments for international trade, for risk pathways of pathogen introduction between farms to inform risk-based surveillance, for modelling of disease transmission, and for the assessment of potential sources of pathogen in an outbreak investigation.

To review the literature on this topic, we assumed the following definitions: vertical transmission means the transfer of infection from parents to progeny through infection of the fertilized egg by the pathogen. The eggs become infected during development in the ovaries or when penetrated by contaminated or infected sperm. In contrast, egg surface-associated transmission means the transfer of infection from parents to progeny through contamination of the egg surface with the pathogen.

IHNV was shown to adhere to sperm under experimental conditions [126]. Some studies (e.g. [127]) report egg associated transmission of IHNV, however without providing sufficient detail or evidence to discriminate whether the transmission to progeny was via vertical or surface associated transmission. On the other hand, fry hatched from eggs of IHNV-positive female rainbow trout did not become infected, when eggs were incubated in IHNV-free water [121]. IHNV could not be detected in masu and chum salmon eggs injected with a dose of 10^3.75^ TCID_50_ IHNV shortly after fertilization by 1 week (after injection masu salmon eggs), and by 5 weeks after infection (in chum salmon eggs), suggesting that IHNV is unlikely to survive within eggs [128]. The results of the latter study suggests that true vertical transmission is unlikely. However, there are also studies demonstrating IHNV infection in progeny of infected broodstock after disinfection of the egg surface: IHNV outbreaks were observed in first feeding fry in 2 out of 7 years in progeny derived from natural summer runs of steelhead trout from a river known to have IHNV-positive fish populations. Eggs were disinfected with an iodophor at water hardening (100 ppm for 1 h) and at the eyed egg stage (100 ppm for 10 min). Hatchery water source came from deep wells. The authors suggested that viral titres may have been so high in ovarian fluid that disinfection protocols were insufficient [129]. Further evidence that disinfection protocols of salmonid eggs are not always effective was provided by another study: Disinfection with 100 ppm iodophor for 60 min at 10 °C did not result in complete inactivation of IHNV on experimentally infected green and eyed rainbow trout eggs [130]. Eggs had been exposed to initial titres of 1.8–8.5 × 10^6^ pfu mL^−1^ IHNV (mimicking an exposure scenario of high viral titres in ovarian fluid) for 60 min. Viable virus titres did decrease by more than 99.98%, however, final titres were still in the order of 10–10^4^ pfu mL^−1^.

Although egg disinfection is not 100% reliable, it is very effective in reducing the likelihood of transmission by IHNV, and it is used routinely in many production facilities [51].

Quantitative estimates for the probability of transmission of IHNV via eggs were provided through an expert consultation. Experts estimated that under the assumption that the source site was sub-clinically infected, of 100 consignments of non-disinfected rainbow trout eggs, 30 would lead to infections of IHNV at receiving sites. If eggs were disinfected, the experts estimated that 5 out of 100 egg consignments would lead to infections of IHNV at receiving sites [35].

The information provided through studies cited above suggest that it is likely that any reports of IHN outbreaks in progeny are the result of inadequate disinfection of eggs that came from broodstock that were likely infected (but not tested) or known to be moderately or severely infected. It appears that disinfection protocols need to consider the health status of broodstock. Water inputs also need to be addressed as well as other potential sources of virus introduction such as equipment and people. More than one standardized protocol may need to be developed to address common practices of salmonid egg producers.

## Diagnostic test performance

Timely diagnostic confirmation of the infectious status of individuals and populations is key to any ability to control disease outbreaks and the spread of infectious diseases. Methods recommended by the OIE for diagnosis of clinical IHN disease are isolation in cell culture followed by identification using a serological method [neutralisation, enzyme linked immunosorbent assay (ELISA), indirect fluorescence antibody test (IFAT)] or a molecular biology method (RT-PCR, DNA probe or sequencing). Alternatively, any two of the following tests: antibody-based assays, DNA probe or RT-PCR can be used. RT-PCR should always be followed by sequencing to confirm the identity of the amplicon [16]. The method recommended by OIE for surveillance for the virus is isolation in cell culture followed by identification using a serological or molecular biology method [16].

In Europe the recently released EU Commission Decision 2015/1554, including Annexe 1 on “Surveillance and control methods” and Annexe II on “Detailed diagnostic methods and procedures” includes in Part 1 of each of these Annexes (concerning VHS/IHN), the use of either RT-qPCR or virus isolation (with confirmation) as suitable methods with equal weight.

The following paragraph summarises recommended sampling and testing procedures in Chapter 2.3.4 on IHN of the OIE Manual of Diagnostic Tests for Aquatic Animals 2015 [16]. Samples for surveillance should be taken when water temperatures are below 14 °C or at their annual lowest if above this. Recommended organs for testing are anterior kidney, spleen and heart or brain. For surveillance ovarian fluid and milt should be examined at least once annually. Samples can be pooled up to a recommended maximum of 10 fish. The recommended cell lines for virus isolation are EPC or FHM. Where relevant, inocula should be pre-incubated with antisera capable of neutralising infectious pancreatic necrosis virus (IPNV). Cell cultures should be inoculated at 1:100 and 1:1000 dilutions and incubated at 15 °C for 7–10 days with regular monitoring for cytopathic effect (CPE). In the absence of CPE sub cultures are made in the same cell line and incubated for a further 7–10 days. There are a number of primer sets published for the identification of IHNV, and recommended primers are published in the OIE manual.

In cases where there is suspicion of subclinical disease or sampling populations that may be recovering from disease there is strong evidence that brain is an important sample for detecting persistent infection [103, 131].

A number of studies have compared virus isolation (VI) with RT-PCR, for example [34, 42, 102, 132–136]. Barlic-Maganja et al. [132] used the same viral dilutions for virus isolation and RNA extraction and molecular detection (dilutions from 10^−1^ to 10^−7^) and did not find molecular methods to be more sensitive compared to cell culture [132]. McClure et al. [136] compared test performance of VI and RT-PCR (nested assay using a different primer set compared to Bergmann et al. [133] and Miller et al. [34]) using Atlantic salmon samples collected in the field. The authors found the operating characteristics (sensitivity and specificity) of RT-PCR were very similar to those of VI and suggested it could be used for field testing fish for IHNV. Samples were tested before and after freezing and the authors reported that there were more positives by VI after freezing; storage of samples in RNAlater^®^ reduced the number of positive samples determined by RT-PCR compared with testing fresh tissue or tissue frozen without RNAlater^®^. Dhar et al. [42] investigated the use of a non-lethal sampling of pectoral fin tissue as a means of diagnosing IHNV. They used SYBR green real time RT-PCR assays to detect the N gene or G gene of IHNV in comparison with a plaque assay for IHNV using fin samples from apparently healthy rainbow trout and rainbow trout showing clinical signs of IHN. The assay for the G gene was more sensitive than that for the N gene and detected IHNV in 33% of the apparently healthy fish and in 67% of the fish showing clinical signs of IHN. That compares with 17 and 92% positive for the two groups of fish by plaque assay. The authors suggested that the higher numbers of clinically infected fish by plaque assay may have been because there may have been more than one virus strain in the field samples and the inability of the primer sets used to detect all such strains [42]. Purcell et al. [136] developed a universal RT-real time PCR to detect IHNV from a wide selection of geographic regions. The test was reproducible in different laboratories, and trials with field samples compared VI with the RT-rPCR. IHNV was detected in 10% of kidney samples using virus isolation compared with 70% in the RT-rPCR. No samples were VI positive but RT-rPCR negative.

In three other papers, detection by RT-PCR appeared to be superior to VI. Miller et al. [34] reported that RT-PCR was the more sensitive method as VI failed to detect 4 out of 7 farms as infected with IHNV, whereas all farms were correctly diagnosed using RT-PCR. Bergmann et al. [133] applied the tests to experimentally infected rainbow trout and found that RT-PCR was significantly more sensitive compared to cell culture. The authors included several IHNV isolates in the study. Hostnik et al. [135] investigated the effect of storing tissue samples and observed that RT-PCR was less affected by storage of samples compared to VI. A slightly higher sensitivity of RT-PCR was also confirmed by Knusel et al. [102].

Sampling regimes, including the required number of fish to be tested, are different according to the purpose of the testing—for example for diagnosis to confirm suspicion of reported clinical cases, for surveillance to determine prevalence or for inspection programmes to determine freedom from infection. The numbers sampled need to reflect population size and epidemiological units and typically larger sample sizes are required for surveillance and inspection programmes. Pooling of samples is often used as an approach when animals are either too small to test individually (e.g. fry) or more often to reduce the cost of testing programs. The OIE manual [16] currently recommends a maximum of 10 samples can be pooled for analysis by virus isolation, whereas the EU diagnostic manuals recommend a maximum of 10 for virus isolation but only five for RT-PCR based tests. These recommendations are not well supported by published references and indeed there is little work in the literature to evidence the effect of sample pooling on detection of IHNV. It is therefore generally recommended that laboratories undertake their own experiments to determine the effects of pooling on test sensitivity with the specific diagnostic tests they use. Most published diagnostic tests have undergone some test validation in order to satisfy peer review at the least. This is generally at the analytical level of test sensitivity (ASe) using known analytes of differing known concentration and specificity (ASp) across a range of related and unrelated pathogens in the laboratory. Diagnostic validation, referring to the assessment of the performance of a test on the real life samples for which it was intended, involves the assessment of a tests ability to correctly determine known positives [(diagnostic sensitivity (DSe)] and known negatives [(diagnostic specificity (DSp)] and thus its predictive value. Diagnostic test performance is a key factor determining the usefulness of inspection and surveillance programmes utilising a particular assay but in the aquatic animal field such diagnostic validation is rarely fully completed to OIE guidelines [19], partly because of the difficulty of obtaining funding for such work and logistically obtaining the required reference samples. IHN is one of the diseases for which some of the diagnostic tests are reasonably well supported by both analytical and diagnostic validation, possibly because of the long history of significant disease outbreaks in important natural populations in North America and associated hatchery reared restocking before the general rise in aquaculture of the 1970s and 80s. Even so there are very few publications on test validation for IHNV. McClure et al. in 2008 [135] validated the performance of virus isolation and the then current (2006 revision) OIE recommended RT-PCR assay (based on the N gene). Analysing 50 fish each from farms with high, low and no prevalence of infected fish (a limited number of samples) the operating characteristics of both assays were similar but with each missing over 10% of positives (DSe of 74–89%). Diagnostic specificity ranged from 92 to 100%. The authors recommended a larger sampling size to better validate this assay. The current RT-PCR assay recommended by the OIE (2012 revision) and EU diagnostic manuals is based on the G gene [137] but the reviewers were not able to find published validation for this assay. Real time PCR is rapidly taking over from conventional PCR in many diagnostic laboratories due to its faster turnaround and comparable sensitivity to a nested conventional PCR but with lower risk of contamination. Purcell et al. [136] recently developed and validated a reverse transcriptase real time PCR assay capable of targeting the N gene and capable of detecting all known IHNV genogroups . Diagnostic validation was undertaken on sample sizes of 50 positive and negative laboratory challenged steelhead trout samples and 60 juveniles from a hatchery undergoing a disease outbreak. The assay was compared to virus isolation and a single round conventional PCR (targeting a different gene) and proposed to be superior at the level of DSe (100% compared to 84% for virus isolation). All three methods had equal and full specificity (DSp of 100%). The real time PCR assay method is now included in the recently released EU diagnostic manual, but is yet to be included in OIE diagnostic manual.

## Pathogen load in fish tissues

Information on pathogen load is of particular relevance for import risk assessments via imported aquatic animal products derived from susceptible species, e.g. for human consumption or used as angling bait. Furthermore, knowledge of pathogen load in tissues is relevant for assessing the potential of pathogen release from mortalities left in fish rearing units and the potential of spread via movements of mortalities (e.g. where mortalities are moved to other farms for storage). The information is also relevant to inform best sampling approaches for diagnostic testing purposes.

Very little information is available on viral load in tissues imported for human consumption (i.e. muscle tissue, head tissues). In most studies, tissues had been frozen or stored prior to analysis. Therefore, the initial viral load is unknown. There was no common method with regards to sample preparation prior to analysis, time of storage, or storage temperature such that the results from the various studies cannot easily be compared. Also, the source of samples varied from fish sampled during naturally occurring outbreaks, infected by bath challenge, or infected by intraperitoneal injection. A summary of the available data is presented in Additional file 1. Values reported from carrier fish are clearly limited by the sensitivity of tests available for the detection of IHNV in tissues. It is quite possible that lower virus titres are present, but the theoretical limit of detection of virus isolation protocols is 1 × 10^2^ virus particles per g tissue input and sampling and analytical procedures (e.g. pooling, sub-standard storage) often mean this is not obtained and therefore lower virus concentrations would escape detection.

The viral load in clinical IHN disease can be very high, regularly up to 10^8^–10^9^ pfu g^−1^ tissue and occasionally 10^10^ pfu g^−1^. However, the average titres are in the order of 10^4^–10^6^ pfu g^−1^. Although several studies have determined the viral load in gills and brain, only one study has evaluated IHNV levels in the skin and none have done so for muscle. Of note are studies reporting detection of IHNV in mucus, in one report at up to 4 × 10^8^ pfu g^−1^. Although high titres of IHNV can be detected in fish during disease outbreaks, the same is not so in surviving fish, in which virus cannot usually be detected. The conclusion is that virus is not present in those fish, or is present in a form or at level that cannot be detected by current analysis methods.

In the text below, we focused on the literature available for rainbow trout, since rainbow trout is the species of greatest importance in Europe in freshwater aquaculture in relation to transfer of IHNV. There are relatively few data on pathogen load in rainbow trout following natural infections, particularly of commodity-size fish, and far more data from experimental infections. Some of the data are derived from fish undergoing clinical disease, whereas the levels of virus found in apparently healthy fish are of greatest relevance to this review.

In naturally infected rainbow trout the level of virus in internal organs ranged from 10^1.7^ to approximately 10^8^ pfu g^−1^ which was similar to that in other species and in experimental infections. High titres of virus (10^7^ pfu g^−1^) can be detected in brain or gill tissues during clinical disease, but the average titres in the gill of virus carrier fish was 10^2.5^ pfu g^−1^ (Additional file 1).

There are no data for level of virus in brain of carrier fish except for spawning and pre-spawning sockeye salmon, in which levels in the brain varied between 10^2^ and 10^3^ pfu g^−1^, compared with 5.4 × 10^3^–3.8 × 10^6^ pfu g^−1^ in the gill and 4.7 × 10^6^ pfu g^−1^ in the spleen. Similar results have been obtained in other studies of spawning sockeye salmon [46, 79, 89, 119, 127]. There has been only one study in which the titre of virus was determined in skin (fin) tissue [138]. Virus was detected in fin tissue from 4 of 24 apparently healthy rainbow trout sampled during a disease epizootic at levels between 1.0 × 10^2^ and 4.0 × 10^4^ pfu g^−1^ and in clinically diseased fish at levels between 1.0 × 10^3^ and 2.14 × 10^6^ pfu g^−1^ [42]. Levels of virus in muscle tissue have not been recorded. IHNV has been detected (10^2.2^–10^2.7^ pfu g^−1^) in mucus of adult carrier rainbow trout and Chinook salmon [118]. The amount of virus was almost double that in spawning kokanee [118], and in moribund commercial size Atlantic salmon was 10^3.3^ pfu g^−1^ (G S Traxler cited by Evelyn [139]). In experimentally infected rainbow trout fry the amount of virus detected in mucus was 1 × 10^4^–4 × 10^8^ pfu g^−1^ [38].

LaPatra et al. [138] assessed the risk of transferring IHNV with commodity rainbow trout (225–500 g). The fish exhibited spinal curvature or spinal compression and were considered to have had high likelihood of previously being infected with IHNV. The fish were negative for IHNV by virus isolation and nested reverse transcription polymerase chain reaction, but, there was no direct evidence that these fish had initially been infected with IHNV. LaPatra et al. [138] also experimentally infected groups of 100 rainbow trout (mean weight 100 g) with IHNV and sampled survivors at weekly or biweekly intervals starting 34 days post infection (10 days or more following the last mortality). The only occasion in which virus was detected in kidney or brain was in one fish sampled 34 days post infection. The authors concluded that IHNV was cleared from the previously exposed rainbow trout.

The information provided above and in Additional file 1 shows that clinically infected fish can carry very high titres in all tissues tested and that also subclinically infected fish can carry significant virus levels. Survivors may possibly fully clear infection (although see Sect. 10), although virus level could also possibly fall below detectable levels rather than be completely cleared from hosts.

## Survival of IHNV in fish tissues

Data for pathogen load in aquatic animal tissues need to be interpreted in combination with information on the persistence of the virus in these tissues. We explained the relevance of knowledge on pathogen load in the previous section (e.g. for analysis in import risk assessments, and design of sampling for diagnostic tests). The information presented here complements the previous section.

The data for survival of IHNV in fish tissues were often obtained from studies to determine parameters relevant to diagnosis of the disease, and so many of the data were obtained for virus survival in internal organs, brain tissue or fry. Most of the data were obtained from experiments in which fish material was seeded with virus. The data from individual trials are summarised in Table 2. The survival of virus in muscle was only determined in one trial in which a homogenate of muscle and skin from fingerling rainbow trout (minus heads, tails and viscera) were seeded with 10^5.7^ TCID_50_ mL^−1^ IHNV [140]. At 4 °C, the most likely holding/shipping temperature of fillets, 10^2^ TCID_50_ mL^−1^ virus survived storage for 7 days. At that temperature the virus survived for 3 weeks in an extract of whole fry but it survived longer in individual organs; up to 4 weeks in liver and 5 weeks in brain. In several studies in which a range of temperatures was compared, the survival of virus was inversely proportional to the temperature. Inactivation of the virus suspended in cell culture medium or tissue extracts at different temperatures was similar at a particular temperature in both matrices, except that at 28 °C there was greater inactivation of IHNV in the tissue homogenate [141]. The authors of the study suggested that this was because 28 °C was near the optimum for the enzymes acting on the virus that had been released during homogenisation.

**Table 2:** Survival of infectious hematopoietic necrosis virus in fish tissues

Species	Tissue	Source of virus contamination	Test conditions (°C)	Starting titre	Titre reduction (log10)/virus detection	Reference
Rainbow trout	Homogenate of kidney, spleen and heart	7–9 cm fish from a farm with a recent history of IHN	4	10^4.8^ TCID_50_ mL^−1^	Titre reduced by about 1–2 log per day. Virus survived 3 (but not 4) days	[134]
			25		No evidence of virus after 1 day	
Rainbow trout	Homogenate of muscle and skin from fingerlings (fish minus heads tails and viscera)	Seeded with IHNV	4	10^5.7^ TCID_50_ mL^−1^	Titre reduced by 3 logs after 7 days	[140]
			10		Titre reduced by 4 logs after 5 days	
			21		Titre reduced by 3 logs after 4 days	
Rainbow trout	Individual homogenates of brain, liver, kidney and spleen from 3 fish of approx 900 g	Seeded with IHNV	−20	10^3.6^–10^3.9^ pfu mL^−1^	Virus survived for 5 months in brain, 3.5 months in liver and spleen and 4 weeks in kidney tissue	[150]
			4		Virus survived at least 5 weeks in brain, 4 weeks in liver, 3 weeks in spleen and was detected after 3 but not 7 days in kidney tissue	
			20		Virus survived at least 1 week in brain, losing 1 log titre; survived 1 day in liver or spleen. Virus detected intermittently over a 3 day period in kidney, but not after 1 week	
Sockeye salmon	Fry	Seeded with IHNV	−20	10^3.7^ pfu mL^−1^	Virus survived 4 weeks	[150]
			4		Detected after 3 but not 4 weeks	
			20		Detected after 4 h but not 24 h	
Sockeye salmon	Homogenate of gut, pyloric ceca, kidney, spleen and liver	Seeded with IHNV	38	>10^7^ pfu mL^−1^	c. 5 logs in <50 min	[141]
			32		c. 4 logs in 2.5 h	
			28		c. 4 logs in 2.5 h	
			8		No significant loss after c 3 h	

The data summarised in Sects. 16 and 17 highlight that IHNV can be present in high concentrations in fish tissues and fish mucus and that cold storage extends the survival of the virus. Where fish in incubation of disease are harvested, these fish could carry significant virus levels. The risk of transmission to naïve fish populations then depends on overall virus quantities released, e.g. with liquid processing waste or when used as predator bait. Of particular concern is processing of potentially infected fish on fish farms.

Experts considered on-site processing an important risk for IHNV introduction. They estimated that out of 100 farm sites receiving rainbow trout carcases for processing from other farm sites (of unknown infection status), five would become infected over a 12-month period. When the processing site received live infected fish, the estimate increased to 77. The risk of infection from the presence of a fish processing facility within 5 km upstream was rated similar to the presence of wild fish populations or stocked fisheries (susceptible species) within 5 km upstream, for which estimates was that over a 1 year period, out of 100 independent farm sites (located in independent water catchments/sea water zones), 10 farm sites become infected. Receiving and storing fish waste (mortalities and processing waste) from other fish farms was considered to carry higher (1.5-fold) risk compared to mechanical transmission of IHNV through staff working on other fish farms (or staff from other fish farms working on the site) [35].

The relevance of potential introduction of aquatic animal viruses for potential pathogen spread was recognised by the World Organisation for Animal Health, OIE and led to a revision of all code chapters for aquatic animal pathogens [19, 142]. As a result, eviscerated carcasses are no longer on the list of products that can be imported into disease free area without health certification.

## Vectors of IHNV

A wide range of farmed fish from freshwater and the northern European marine environment, and to a much lesser degree farmed marine Mediterranean fish, are considered possible vectors of IHNV. Furthermore, there is evidence for the potential of IHNV transmission via invertebrates and piscivorous birds, and other animals may play a role.

A vector is typically defined as an organism that transmits a disease agent from one animal or plant to another. However, in a study to compile a list of vector species in aquaculture, the European Food Safety Authority (EFSA) narrowed and limited the definition of a vector species to (1) an animal that was farmed (2) that was traded live for farming purposes and (3) was non-susceptible to the disease in question [143]. That was in order to determine which vector species could be introduced for farming or restocking purposes into a specific disease-free zone of a member state of the European Union. [143]. Based on those criteria and under specific conditions (the vector species are sourced from the farm which keeps a susceptible species and are introduced to a new site with a susceptible species), members of the *Acipenseridae*, *Cyprinidae*, other freshwater fish (non-*Cyprinidae*), marine northern European fish and freshwater crustaceans were judged to have a moderate likelihood of being vectors of IHNV and marine Mediterranean fish a very low likelihood. If those specific conditions did not apply, the likelihood of those animals being vectors of IHNV was negligible [143] (see Sect. 10).

IHNV has been isolated from a number of invertebrates. IHNV was isolated from leeches (identity not given) collected from spawning sockeye salmon on three sampling periods at approximately 1-week intervals. Leeches positive for IHNV increased from 67 to 100% over the 3 weeks, and on one occasion the virus titre was >10^6^ pfu g^−1^ [89]. In another study IHNV was isolated from leeches (*Piscicola salmositica*) and a copepod (*Salminicola* sp.) both parasitizing sockeye salmon. Average virus titres in pools of the copepods were 7.8 × 10^3^ pfu g^−1^ and the range of titres in individual leeches was 2.5 × 10^1^–8.7 × 10^5^ pfu g^−1^ [144]. There was no evidence that the virus replicated in the leeches. IHNV was isolated from adult Mayflies (*Callibaetis* sp.) collected from streams and an abandoned fish hatchery on a number of occasions, but the titre of virus was not determined [145].

Although isolation of IHNV from the invertebrates suggests that they may be vectors of the virus, evidence is sparse. One study [146] showed that the salmon louse, *Lepeophtheirus salmonis*, could transmit the virus in the laboratory. Although salmon lice are often considered not to transfer between hosts, such transfers have been observed under farmed and laboratory conditions, particularly when the host fish were kept at high densities [146, 147], and so this mode of transmission is feasible in aquaculture under certain circumstances. Lice exposed to 1 × 10^5^ pfu mL^−1^ IHNV in water and lice that parasitized Atlantic salmon experimentally infected with IHNV acquired the virus and those acquiring it from infected Atlantic salmon remained virus positive for 12 h. In further experiments, lice that were exposed to IHNV in water or had parasitized experimentally infected Atlantic salmon were put in different tanks containing naive Atlantic salmon. Mortalities of 66.6 and 70.6% were observed in the two tanks of fish respectively, and IHNV was recovered from the majority of exposed fish. The authors concluded that under the experimental conditions the lice transmitted the virus to the fish, and transmission was likely to be mechanical rather than biological.

Experts estimated the number of farms (out of 100) becoming infected with IHNV over the course of 1 year due to short distance mechanical transmission (defined as introduction of pathogen from sources in close proximity to the farm through routes including: piscivorous birds or other animals and assuming no direct water connectivity). The experts estimated that one farm (or two farms) would become infected when assuming a country wide farm level prevalence of 2% (or 5%) in a hypothetical country [35].

## Conclusions

We have reviewed the peer reviewed literature to summarise the information relevant for a range of purposes, e.g. the preparation of import risk assessments; the parameterisation of pathogen spread models; for surveillance planning, including risk-based surveillance; to evaluate the chances of eradication of the pathogen.

To provide an example on using information presented in this review for surveillance planning:

If the purpose was surveillance to demonstrate freedom from IHNV in a defined geographic area (freshwater) in Europe, the sampling should (1) target species most likely to develop clinical infection if infected (e.g. rainbow trout); (2) target a life stage that is most likely to develop clinical disease if infected (i.e. fry, or adult fish post spawning); (3) the water temperature at which sampling should be undertaken is ideally 10–12 °C; (4) if multiple rearing units fulfil the above criteria, animals that have been, or currently are subject to stressful conditions (e.g. recent grading, or poor water quality such as low oxygen levels) should be preferentially selected; (5) fish kept at high rearing density should be preferentially selected (since this may increase the chance of fish to fish transmission). Once rearing units meeting the above criteria have been identified, fish showing clinical signs of disease within these should preferentially selected. In contrast, sampling of non-stressed adult fish reared at low stocking density is most likely to return a negative test result, which does not mean though that the farm population is not infected.

Examples of factors to consider in models for IHNV spread via live fish movements are: species moved; size of the consignment; prevalence of infection in the consignment; rate of virus shedding by moved fish; host susceptibility parameters of fish at receiving farm (to estimate likelihood of infection establishing at receiving farm).

Significant variability in outcome of pathogen exposure was found for almost all factors that influence IHNV infection. For example, mortality levels may vary significantly depending on fish strain, virus exposure levels, water temperature—to name just a few. Modelling of pathogen transmission will therefore need to allow for fairly broad confidence levels. One aspect that is highly relevant for modelling the early phase of IHNV spread within a fish population, is that stressful events, such as netting of fish, or live fish movements, are likely to lead to mucus shedding. Mucus of infected fish may contain very high levels of IHNV. One study found an immediate (within 1 min) rise of virus concentration in rearing water, after fish were transferred into a new container [43]. The consequence of such sudden bursts of virus release is that the minimum infectious dose could quite possibly be exceeded as a result.

The variability in outcome of pathogen exposure should also be considered in the design of surveillance programmes and the interpretation of test results from diagnostic samples.

The variability in outcome of virus exposure has implications for expected prevalences within susceptible fish populations and diagnostic test sensitivity (the likelihood of a diagnostic test to correctly identify an animal as infected, if infected). Knowledge of the diagnostic test sensitivity is relevant for surveillance planning, interpretation of data from disease investigations, evaluation of the chances of eradication, and also for the interpretation of many of the data presented in this review. With increasing size and age, some susceptible fish species (e.g. rainbow trout) appear less likely to be clinically affected and as a result the chance of detection of the virus is reduced. The consequence of failing to detect infection could be significant, since fish from such farms could be permitted to be moved live into declared disease free areas, despite carrying the virus.

Related to the above is the need for more information on subclinical infection, including for how long carrier status may persist. For example, to assess the likelihood of transmission of IHNV from subclinically infected to naive fish, information required (at a minimum) is: (1) knowledge about prevalence in subclinically infected populations; (2) the quantity of virus discharged from such fish per time unit; and (3) the minimal infectious dose for exposed species. Different challenge setups (e.g. longer exposure times in bath challenges) may be required to imitate realistic conditions in the field.

The majority of studies reviewed here were undertaken on the major salmonid species produced in aquaculture (Atlantic salmon and rainbow trout) and, with regards to wild fish, on sockeye salmon. Further information is urgently required on the risk of spread of IHNV via other commercially traded species (e.g. brown trout, Arctic char and grayling). Lack of data means that significant risks (e.g. risk of spread via live fish movements) may not be recognised and that international legislation possibly omits species that should be listed as susceptible, allowing trade of these species without conditions on IHNV health status. Live fish movements of fish species not listed as susceptible to IHNV under EU Directive 2006/88 played a role in IHNV epidemic in Germany in 2006, which led to a spread of the disease in 5 farms (Nardy, personal communication March 2016; [148]). Not listing species scientifically shown to be susceptible to IHNV in regulations controlling international trade for disease control purposes is significant as the industry may increasingly move to farming species not covered by the legislation, which could increase the risk of pathogen spread, as potentially infected fish would not be subject to requirements regarding their IHNV health status.

Reasons for lack of data in certain areas may possibly be due to limited interest within the scientific community or from funding organisations for such studies, as they don’t always require advanced techniques to deliver results. However, the value of generating such data is significant.

One particular concern that arises from the review is that IHNV may affect the Atlantic salmon aquaculture industry beyond the Pacific coast of North America. The cause for emergence of IHNV in Atlantic salmon in North America was the introduction of a new species into an environment where IHNV was endemic in wild fish. As IHNV is not currently endemic in wild marine salmonid species outside of its natural range at the west coast of North America, the risk posed by IHNV is from a possible introduction of the pathogen via other pathways into farmed or wild fish populations with a possible establishment of IHNV in an endemic infection. The potential for wild Atlantic salmon populations to support IHNV in an endemic infection is currently unknown. The reviewed data suggest that IHNV could be a significant threat to the Atlantic salmon industry in Europe and other geographic areas in which Atlantic salmon are farmed.

## Additional file

10.1186/s13567-016-0341-1 **Infectious hematopoietic necrosis virus pathogen load in fish tissues.** Table with IHN virus concentrations in a range of tissues from a variety of susceptible fish species.
