# Supplementary material for: Epidemiological characteristics of infectious hematopoietic necrosis virus (IHNV): a review
Source: Vet Res. 2016 Jun 10;47:63. doi: 10.1186/s13567-016-0341-1 (PMC4902920; doi:10.1186/s13567-016-0341-1)
Supplement: Supplementary file 1 — 10.1186/s13567-016-0341-1 Infectious hematopoietic necrosis virus pathogen load in fish tissues. Table with IHN virus concentrations in a range of tissues from a variety of susceptible fish species. [file 13567_2016_341_MOESM1_ESM.docx]

**Dixon et al. (2016) Epidemiological characteristics of infectious hematopoietic necrosis virus (IHNV). Veterinary Research. DOI 10.1186/s13567-016-0341-1**

**Additional file 1. Infectious hematopoietic necrosis virus pathogen load in fish tissues.**

| **Species** | **Pathway of infection** | **Sampling** | **Tissue sampled** | **Virus concentration (TCID50)* g-1 tissue** | **Virus concentration (PFU)** g-1 tissue or mL-1 fluid** | **Reference** |
| --- | --- | --- | --- | --- | --- | --- |
| Rainbow trout (steelhead) | Natural infection, carrier fish | Spawning male | Spleen |  | 10^1.7^ - 10^8.08^ | [[118](#_ENREF_118)] |
|  |  |  | Kidney |  | 10^2.18^ - 10^7.9^ |  |
| Rainbow trout | Natural infection, disease epizootic | Apparently healthy; no other data | Fin tissue |  | 1.0 × 10^2^ - 4.0 × 10^4^ (4/24 fish) | [[41](#_ENREF_41)] |
|  |  | Clinically diseased; no other data |  |  | 1.0 × 10^3^ - 2.14 × 10^6^ (33/36 fish) |  |
| Rainbow trout | Natural infection, disease epizootic | Mean weight 13 g, “hematopoietic” form of disease at start of epizootic | Kidney/spleen |  | 4 × 10^4^ - >2 × 10^7^ | [[130](#_ENREF_130)] |
|  |  |  | Brain |  | 1.1 × 10^5^ - 2.4 × 10^6^ |  |
|  |  | Mean weight 5 g, “neurotrophic” form of disease at end of epizootic | Kidney/spleen |  | Not detected |  |
|  |  |  | Brain |  | 3.6 × 10^5^ - 2.0 × 10^7^ |  |
| Rainbow trout (steelhead) | Natural infection, carrier fish | Adult | Mucus |  | 10^2.6^ (mean concentration) | [[117](#_ENREF_117)] |
|  |  |  | Gill |  | 10^2.5^ (mean concentration) |  |
| Chinook salmon | Natural infection, chronic mortality | Yearling | Nervous tissue |  | 10^2^ - 10^6^ | [[117](#_ENREF_117)] |
|  |  |  | Mucus |  | 10^1.3^ - 10^2.4^ |  |
|  | Natural infection, disease epizootic | Juvenile | Mucus |  | 10^2.5^ - 10^5.8^ |  |
|  | Natural infection, carrier fish | Adult | Mucus |  | 10^2.2^ - 10^2.7^ |  |
| Landlocked sockeye salmon (kokanee) | Natural infection, carrier fish | Spawning female | Mucus |  | 10^4.2^ (mean concentration) | [[117](#_ENREF_117)] |
|  |  | Spawning male | Mucus |  | 10^3.3^ (mean concentration) |  |
| Atlantic salmon | Natural infection, seawater | Commercial size, moribund | Mucus |  | 10^3.3^ | G S Traxler cited by Evelyn [[138](#_ENREF_138)] |
|  |  |  | Visceral tissue |  | 105 |  |
| Sockeye salmon | Natural infection, carrier fish | Prespawning female | Spleen |  | 1.6 × 10^4^ | [[148](#_ENREF_148)] |
|  |  |  | Kidney |  | 6.5 × 10^3^ |  |
|  |  |  | Liver |  | 3.1 × 10^4^ |  |
|  |  |  | Lower gut |  | 1.1 × 10^4^ |  |
|  |  |  | Pyloric ceca |  | 3.0 x 10^4^ |  |
|  |  |  | Gill |  | 5.4 × 10^3^ |  |
|  |  |  | Brain |  | 5.5 × 10^2^ |  |
|  |  | Ripe females | Spleen |  | 2.3 × 10^5^ |  |
|  |  |  | Kidney |  | 1.4 × 10^5^ |  |
|  |  |  | Liver |  | 1.5 × 10^5^ |  |
|  |  |  | Lower gut |  | 4.5 × 10^5^ |  |
|  |  |  | Pyloric ceca |  | 2.4 × 10^5^ |  |
|  |  |  | Gill |  | 2.7 × 10^5^ |  |
|  |  |  | Brain |  | 9.3 × 10^2^ |  |
|  |  | Spent females | Spleen |  | 2.2 × 10^4^ |  |
|  |  |  | Kidney |  | 3.1 × 10^4^ |  |
|  |  |  | Liver |  | 2.2 × 10^3^ |  |
|  |  |  | Lower gut |  | 4.3 × 10^3^ |  |
|  |  |  | Pyloric ceca |  | 1.5 × 10^3^ |  |
|  |  |  | Gill |  | 7.9 × 10^3^ |  |
|  |  |  | Brain |  | 1.5 × 10^2^ |  |
|  |  | Spawning males | Spleen |  | 4.7 × 10^6^ |  |
|  |  |  | Kidney |  | 5.8 × 10^4^ |  |
|  |  |  | Liver |  | 1.1 × 10^5^ |  |
|  |  |  | Lower gut |  | 2.5 × 10^6^ |  |
|  |  |  | Pyloric ceca |  | 2.7 × 10^6^ |  |
|  |  |  | Gill |  | 3.8 × 10^6^ |  |
|  |  |  | Brain |  | 1.1 × 10^3^ |  |
| Landlocked sockeye salmon (kokanee) | Diseases epizootic | 2 years old fish |  |  |  | [[74](#_ENREF_74)] |
|  |  | Dead fish | Kidney |  | 9.0 × 10^5^ - 1.3 × 10^9^ |  |
|  |  | Moribund fish | Kidney |  | 2.9 × 10^8^ - 1.0 × 10^9^ |  |
|  |  | Moribund fish | Gill |  | 2.5 × 10^7^ - 5.5 × 10^7^ |  |
| Brown trout | Disease epizootic in summer steelhead (rainbow) trout | Fingerling, 8 month old (dead) | Pools of gill, kidney and spleen | 1.0 × 10^5^ - 2.8 × 10^8^ |  | [[100](#_ENREF_100)] |
|  |  | Fingerling, 8 month old (live) |  | <10 to 7.9 × 10^3^ |  |  |
| Rainbow trout | Experimental infection, immersion | Fry, average weight 0.6 g, 4-9 days post infection | Whole fry | >10^7^ |  | [[121](#_ENREF_121)] |
| Rainbow trout |  | Fry, average weight 10 g | Kidney |  | 10^2.8^ (2 days p.i.^***^) 10^7.7^ (7-8 days p.i.) <10^2^ (22 days p.i.) | [[38](#_ENREF_38)] |
| Rainbow trout | Experimental infection, immersion | Fry, average weight 1 g | Mucus |  | 1 × 10^4^ - 4 × 10^8^ (6-14 days pi) | [[37](#_ENREF_37)] |
|  |  |  | Whole fry |  | 4 × 10^4^ - 5 × 10^9^ (6-14 days pi) |  |
| Rainbow trout | Experimental infection, immersion | Fry, average weight 1.6 g or 2.3 g | Whole fish, kidney/spleen or brain |  | 10^5^ (mean titre) | [[130](#_ENREF_130)] |
| Rainbow trout | Experimental infection, immersion | 2 month old (sampled after infection) | kidney, pyloric caeca, spleen or heart | 10^4^ - 10^7^ |  | [[130](#_ENREF_130)] |
|  |  |  | Intestine or stomach | 10^3^ – 10^6^ |  |  |
|  |  |  | Liver | 10^4^ – 10^6^ |  |  |
|  |  |  | Brain | 10^3^ – 10^5^ |  |  |
|  |  |  | Gill | 10^3^ – 10^7^ |  |  |
| Rainbow trout | Experimental infection, immersion | 4 month old (dead fish) | Pooled organs | 10^2^ - 10^6^ |  | [[130](#_ENREF_130)] |
| Rainbow trout | Experimental infection, intraperitoneal injection (two studies) | Average weight 100 g, dead fish | Kidney |  | 10^6.9^ and 10^7.1^ (mean titre) | [[137](#_ENREF_137)] |
|  |  |  | Brain |  | 10^5.8^ (mean titre), both studies |  |
|  |  | Survivors (days 34-76 pi) | Kidney  Brain |  | Virus only detected in one study, in brain at 34 days pi |  |
| Chinook salmon | Experimental infection, immersion | Sexually mature females, 4-14 days pi | Gill |  | 1.25 × 10^4^ - 1.78 × 10^7^ | [[75](#_ENREF_75)] |
|  |  |  | Kidney/spleen |  | 5.0 × 10^2^ - 4.68 × 10^5^ |  |
| Brook trout | Experimental infection, immersion | Fry, 3 months old | Dead fish |  | >10^5^ | [[87](#_ENREF_87)] |
|  |  |  | Asymptomatic survivors, 3 weeks pi |  | 10^4.23^ |  |
| Coho salmon | Experimental infection, intraperitoneal injection | Mean weight 9 g | Dead fish (1) |  |  | [[60](#_ENREF_60)] |
|  |  |  | Kidney spleen |  | 3.0 × 10^6^ |  |
|  |  |  | Liver |  | 1.8 × 10^5^ |  |
|  |  |  | Brain |  | 6.0 × 10^3^ |  |

* 50% tissue culture infectious dose

** Plaque forming units

*** Post infection
